# Inhibitors of Microglial Neurotoxicity: Focus on Natural Products

**DOI:** 10.3390/molecules16021021

**Published:** 2011-01-25

**Authors:** Dong Kug Choi, Sushruta Koppula, Kyoungho Suk

**Affiliations:** 1 Department of Biotechnology, Konkuk University, Chungju 380-701, Korea; 2 Department of Pharmacology, Brain Science and Engineering Institute, CMRI, Kyungpook National University School of Medicine, Daegu 700-422, Korea

**Keywords:** microglia, neuroinflammation, neurodegeneration, natural products, neuroprotection

## Abstract

Microglial cells play a dual role in the central nervous system as they have both neurotoxic and neuroprotective effects. Uncontrolled and excessive activation of microglia often contributes to inflammation-mediated neurodegeneration. Recently, much attention has been paid to therapeutic strategies aimed at inhibiting neurotoxic microglial activation. Pharmacological inhibitors of microglial activation are emerging as a result of such endeavors. In this review, natural products-based inhibitors of microglial activation will be reviewed. Potential neuroprotective activity of these compounds will also be discussed. Future works should focus on the discovery of novel drug targets that specifically mediate microglial neurotoxicity rather than neuroprotection. Development of new drugs based on these targets may require a better understanding of microglial biology and neuroinflammation at the molecular, cellular, and systems levels.

## 1. Introduction

Inflammation in the brain and the rest of the central nervous system (CNS) is a key factor in neurodegenerative diseases. Multiple lines of evidence suggest that microglia, the resident immune cells of the CNS, play a critical role in inflammation-mediated neurodegeneration. Normally, microglia cells in their resting state vigilantly monitor the health of neurons and have a benevolent effect. In the event of brain damage or infection, microglia cells become activated and may secrete a variety of inflammatory mediators and neurotoxic factors. In this condition, activated microglia cells trigger and maintain an inflammatory response, deluging neurons with a whole host of inflammatory mediators that may ultimately lead to neuronal cell death. Neurodegenerative CNS diseases, including Alzheimer’s disease (AD), Parkinson’s disease (PD), Huntington’s disease (HD), amyotrophic lateral sclerosis (ALS), tauopathies, and age-related macular degeneration (ARMD), are all associated with chronic neuroinflammation and elevated levels of several cytokines. Microglial activation and chronic inflammation thereafter is the starting point for elevated levels of a wide array of potentially neurotoxic molecules including pro-inflammatory cytokines, proteinases, and reactive oxygen species (ROS) [1,2,3,4,5,6], which are believed to contribute to neurodegenerative processes [7,8]. Several methods have become available for identifying activated microglia, and their presence has been demonstrated in a variety of neuroinflammatory/neurodegenerative diseases such as AD, PD, ALS and multiple sclerosis (MS) [9,10,11]. This participation of activated microglia and the release of neurotoxic products in the demise of neurons have now been postulated in most, if not all, neurodegenerative diseases. 

A corollary of neuroinflammation proposes that suppression of microglial production of neurotoxic mediators will result in neuroprotection. This heightens the interest in the rapid discovery of neuroinflammation-targeted therapeutics. Efforts include the isolation of natural products and their active components and the screening of existing CNS drugs approved for other uses, as well as the development of novel synthetic compounds that selectively downregulate neuroinflammatory responses. Here, we review recent literature on natural products-based inhibitors of microglia-mediated neurotoxicity. 

## 2. Natural Products and Their Active Constituents as Inhibitors of Microglia-Mediated Neurotoxicity

In recent years, many herbal plants and their active components have emerged and have been subjects of extensive research. These medications have been validated by traditional usage and are time-tested, as compared to modern day trendy supplements. The traditional herbal medicines with dependable ethnopharmacological properties have recently been demonstrated to possess neurotrophic and neuroprotective abilities, which can be useful in preventing various forms of neuronal cell loss in neurodegenerative and neuroinflammatory diseases. During the last two decades, several ingredients from natural products have been tested for their inhibitory actions on neuroinflammation and used as aids to improve memory, treat neurodegenerative diseases or create favorable effects on the CNS. Numerous botanicals have been shown to provide anti-inflammatory and antioxidant activity, which may protect the brain from inflammatory damage. Increasing evidence suggests that traditional herbal extracts possess neuroprotective benefits through distinct and multiple mechanisms, including anti-inflammation [12,13,14,15,16,17]. Natural compounds specifically aimed at blocking microglial activation may be more efficacious at ameliorating microglia-associated neurodegenerative and neuroinflammatory diseases. In the following sections, we will focus on popular and important natural products and their active constituents as well as their anti-inflammatory activities based on inhibiting microglial activation.

## 3. Ginsenosides from *Panax ginseng*

Ginseng, part of the Araliaceae family and species in the genus *Panax*, is found throughout the world. The name *Panax* means ‘‘all healing,” which describes the traditional belief that ginseng has properties to heal all aspects of the body. Ginseng is prepared and used in several ways: as fresh ginseng (sliced and eaten, or brewed in a tea), white ginseng (peeled and dried), or red ginseng (peeled, steamed, and dried). Traditional medicine suggests that red ginseng is the most potent, but modern research has shown that all forms have many beneficial properties [18,19,20]. Mechanisms include inhibition of DNA damage [21], induction of cancer cell apoptosis [22], and inhibition of cell proliferation [23]. Ginseng has been proved to possess potent chemotherapeutic effects. Ginseng and its active components significantly decrease several types of cancers in the pharynx, stomach, liver, pancreas, and colon [24,25].

There is abundant evidence that ginseng has potent effects on key players in the inflammatory cascade. Ginseng extracts and total saponins significantly suppress NF-κB and MAP kinase activities, which are upstream signaling molecules in inflammation. The ginsenosides Rh2, Rh3 and compound K significantly inhibit lipopolysaccharide (LPS)-induced inducible nitric oxide synthase (iNOS) and cytokine expressions, thereby proving their beneficial effect in various neuroinflammatory diseases. Another recent study revealed that American ginseng selectively inhibits the expression of iNOS via suppression of the STAT cascade in inflamed macrophages [26]. Ginseng also inhibits the LPS-induced tumor necrosis factor alpha (TNF-α) and other pro-inflammatory cytokines produced by cultured macrophages [27]. Recent studies by Park *et al.* showed the beneficial effect of ginseng extract and total saponins on microglial activation in co-cultured murine BV-2 microglia and B35 rat neuroblastoma cells [28].

**Figure 1:**
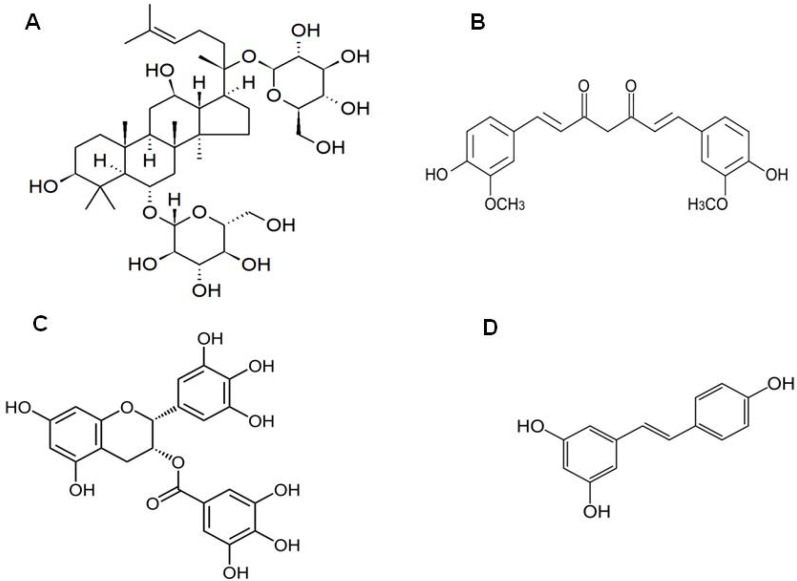
The molecular structure of ginsenoside Rg3 (A), curcumin (B), epigallocatechin-3-gallate (C) and resveratrol (D).

The single compound ginsenoside Rg3 (Figure 1A) inhibited phorbol ester–induced cyclooxygenase-2 (COX-2) and NF-κB induction [29]. Studies also revealed that ginsan, a polysaccharide extracted from *P. ginseng*, inhibits the p38 MAP kinase pathway and NF-κB *in vitro,* and the pro-inflammatory cytokines *in vivo* [30]. BST204, a fermented ginseng extract, can inhibit iNOS expression and subsequent nitric oxide (NO) production from LPS-stimulated RAW264.7 murine macrophages. Ginsenoside Rg3 was also reported to attenuate neuroinflammation in the co-culture of mouse primary dopaminergic neurons and glia. The anti-inflammatory effects of ginsenoside were demonstrated by a reduction in NO formation and PGE_2_ synthesis and involve interference with iNOS and COX-2 expression, making the molecule useful in treating or preventing various neuroinflammatory diseases including PD [31]. Various forms of ginseng have ubiquitous properties to stop inflammation via microglia-mediated mechanisms. Furthermore, intravenous infusion of ginsenoside derivative Rb1 prevented the ischemic neuronal death, and it passed through blood-brain barrier [32]. This natural herb is therefore one of the most important sources for treating various neuroinflammatory diseases. 

## 4. Curcumin from *Curcuma longa*

Curcumin, a yellow pigment compound (Figure 1B), in the widely used spice turmeric, exerts anti-inflammatory activity. Curcumin antagonizes many steps in the inflammatory cascade, including activator protein-1 transcription, activation of nuclear factor-κB, iNOS, and JNK [33,34]. Curcumin seems to be relatively safe, even in clinical trials for prevention of relapse of ulcerative colitis [35]. Recent reports indicate that curcumin protects pre-oligodendrocytes from activated microglia *in vitro* and *in vivo.* Curcumin significantly inhibits the apoptosis of pre-oligodendrocytes and expression of either iNOS or NOX in the LPS-activated microglia. In *in vivo* studies, curcumin decreases activated microglia and inhibits microglial expression of iNOS and translocation of p67phox and gp91phox to microglial cell membranes in neonatal rat brains following LPS injection [36]. Studies have also demonstrated the beneficial effects of curcumin on oxidative damage and amyloid β pathology in a transgenic mouse model of AD. The authors reported that low and high doses of curcumin significantly lowered oxidized proteins and interleukin-1 beta (IL-1β), a pro-inflammatory cytokine elevated in thebrains of these mice [37]. In microglia (rat primary microglia and murine BV-2 microglial cells), curcumin effectively suppressed the ganglioside-, LPS-, or IFN-γ-stimulated induction of COX-2 and iNOS, important enzymes that mediate inflammatory processes. The suppressive effect of curcumin is thought to involve regulating the JAK-STAT inflammatory signaling inactivated microglia [38]. A recent report by Wang *et al.* revealed that curcumin reduces the amyloid-β-stimulated inflammatory responses in primary astrocytes. The deleterious effects of amyloid-β, such as increased expression of COX-2 and glial fibrillary acidic protein and decreased peroxisome proliferator-activated receptor gamma (PPARgamma), were attenuated by pretreatment with curcumin [39].

Accumulating cell culture and animal model data show that curcumin is a strong candidate for use in the prevention or treatment of major disabling age-related neurodegenerative diseases like AD, PD, and stroke [40]. Curcumin administration has been reported to attenuate cognitive deficits, neuroinflammation, and plaque pathology in AD models [41,42,43]. It was also reported that curcumin crosses the blood-brain barrier in an animal model of Alzheimer’s disease when injected peripherally [43]. Further, the outstanding safety profile of curcumin and its pleiotropic actions with potential for neuroprotective efficacy including anti-inflammatory, antioxidant, and anti-protein-aggregate activities achieved at sub-micromolar levels make this compound a potential therapeutic agent in treating neuroinflammatory diseases. 

## 5. Epigallocatechin-3-Gallate (EGCG) from *Camellia sinensis*

One of the greatest achievements in liquid nutrition has come from green tea, a popular drink made from the dried leaves of *Camellia sinensis* (L). It is well known that green tea, one of the oldest beverages in the world, has several beneficial effects in many diseases including cardiovascular disorders, obesity and cancer, in addition to slowing down the process of aging. Therefore, the effect of green tea in the prevention and alleviation of neurodegenerative diseases has been of particular interest in recent years.

The most active component of green tea that shows therapeutic effects is (–)-epigallocatechin-3-gallate (EGCG) (Figure 1C). The green tea polyphenol EGCG inhibits the production of numerous inflammatory mediators, including TNF-α, IL-1β and IL-6 [44]. Furthermore, green tea crosses the blood brain barrier, reduces inflammation, provides antioxidant activity, and reduces neural cell death [45]. EGCG is reported to be significantly more potent than other known antioxidants such as vitamin C and vitamin E in scavenging free radicals [46,47,48]. The neuroprotective effect of EGCG could be achieved through complementary mechanisms involving the down-regulation of pro-apoptotic genes [49,50], the influence of amyloid precursor protein (APP) processing by the elevation of α-secretase activity [51], inhibition of β-secretase activity [52], promotion of cell survival, defense against anti-inflammation [53], oxidative stress [54], scavenging of ROS [55,56], iron chelation, and stabilization of mitochondrial function [57]. 

Aktas and group reported in 2004 that EGCG mediates NF-κB inhibition and exerts neuroprotection in autoimmune encephalomyelitis. The authors suggest that EGCG is capable of protecting against neuronal injury in brain tissue induced by *N*-methyl-D-aspartate or TRAIL and of directly blocking the formation of neurotoxic ROS in neurons [58]. Another report by Li *et al.* indicated that EGCG inhibits LPS-induced microglial activation and protects against inflammation-mediated dopaminergic neuronal injury. The authors suggest that EGCG potently inhibits LPS-activated microglial secretion of NO and TNF-α through the down-regulation of iNOS and TNF-α gene expression. In addition, EGCG exerts significant protection against microglial activation-induced neuronal injury both in the human dopaminergic cell line SH-SY5Y and in primary rat mesencephalic cultures [53]. The antioxidative activity of green tea has also been verified in human clinical trials [59]. Based on the above mentioned reports, green tea, in particular EGCG could be a promising candidate for treating or preventing various neurodegenerative and neuroinflammatory diseases.

## 6. Resveratrol

Resveratrol (*trans*-3,4,5-trihydroxystilbene), a natural polyphenol (Figure 1D), is a phytoalexin present in grapes and various medicinal plants [60]. Studies have documented that resveratrol exhibits a wide range of biological and pharmacological activities, such as anti-carcinogenesis, cardiovascular protection, and anti-inflammatory effects [61,62]. Resveratrol can attenuate the activation of immune cells and the subsequent synthesis and release of pro-inflammatory mediators through the inhibition of transcriptional factors such as NF-κB and activator protein-1 (AP-1) [63]. Earlier published data revealed that resveratrol potently inhibits the production of NO and TNF-α by LPS-activated microglia and suppresses LPS-induced degradation of I-kappa B alpha (IκB-α), expression of iNOS and phosphorylation of p38 MAPKs in N9 microglial cells [64]. Recently, Meng *et al.* evaluated the effects of 21 resveratrol derivatives on LPS-induced NO production in microglia and their structure–activity relationships. The authors reported the potent inhibitory effects of selected compounds on microglial activation, suggesting their potential use for treatment of neurodegenerative diseases accompanied by microglial activation [65]. Moreover, the neuroprotective effects of resveratrol have been extensively studied in several cell lines and primary microglial cultures [64,65,66,67]. Resveratrol has been shown to inhibit the activation of microglia and reduce the production of pro-inflammatory factors through intracellular cascades of signaling pathways such as MAPKs, phosphoinositide3-kinase (PI3-K)/Akt, and glycogen synthase kinase-3β (GSK-3β) pathways. Resveratrol was also neuroprotective by inhibiting the activity of NADPH oxidase signaling pathway. In addition, resveratrol can cross the blood–brain barrier and modulate some of the symptoms of debilitating neurological disorders, such as ischemia [68], PD [69], AD [70,71] and Huntington’s disease [72].

*In vitro* studies have also proved the beneficial effect of resveratrol in various neuronal and microglial cells such as PC12, SH-SY5Y, BV-2 and N9 cells [73,74,75]. It has been noted that resveratrol prevents both Aβ- and MPP^+^-induced PC12 cell death by inhibiting ROS production and caspase-3 and Bax activities, and by up-regulating Bcl-2 activity [73,75]. In human neuroblastoma SH-SY5Y cells, excess dopamine-induced cell death could be inhibited by resveratrol through ameliorating intracellular oxidative stress and enhancing the activity of Bcl-2, thus disrupting the apoptotic machinery [76]. Several studies in the N9 microglial cell line have indicated that resveratrol attenuates LPS-induced phosphorylation of p38 MAPK and degradation of IκB-α, thus reducing the production of NO and TNF-α [64,67].

These studies add to our understanding of the potential mechanisms of resveratrol-mediated neuro-protection against various neurotoxicants through its antioxidant and anti-inflammatory properties. Further, as resveratrol crosses the blood–brain barrier and has a good safety profile, this compound can be an ideal candidate for treating neuroinflammatory and neurodegenerative diseases.

## 7. Gastrodin from *Gastrodia Elata*

*Gastrodia elata* Blume (GE) has been used as a traditional herb and has been considered one of the most important medicinal plants in Oriental countries for centuries. GE has been used for a variety of conditions such as treatment of headaches, dizziness, vertigo, and convulsive illnesses such as epilepsy and tetanus [77]. GE reduces oxygen free radicals [78], protects against neuronal damage [79,80,81], and exhibits anxiolytic-like effects via the GABAergic nervous system [82]. GE inhibits NO production and expression of iNOS and COX-2 upon stimulation by LPS in RAW264.7 macrophages [83]. 

Another study indicated that the ethyl ether fraction of GE dramatically protects neuronal cells from amyloid-β peptide-induced cell death *in vitro* [38], which is closely associated with AD, and has a protective effect against neuronal damage following global ischemia in gerbils [38]. Methanol extract of GE reduces neuronal nitric oxide synthase activity, microglial activation and apoptosis in the kainic acid-treated rat hippocampus [84]. GE also prevents serum-deprived apoptosis of rat pheochromocytoma cells through activation of the serine/threonine kinase-dependent pathway and suppression of c-Jun NH2-terminal kinase (JNK) [85]. Studies conducted in our lab revealed that GE effectively attenuates cytotoxicity and improves cell viability in MPP^+^-induced toxicity in SH-SY5Y cells. GE effectively inhibits both the increased production of ROS and the increase in Bax/Bcl-2 ratio, as well as caspase-3 cleavage and PARP proteolysis. The protective effect may be ascribed to its significant anti-oxidative and anti-apoptotic properties [79].

The major active components in GE are phenolic compounds, gastrodin, *p*-hydroxybenzyl alcohol, vanillyl alcohol, 4-hydroxybenzaldehyde, vanillin, succinic acid, organic acids, glucose, and β-sitosterol [78,86,87,88]. These components play various roles in the treatment of diseases. Recently, GE was reported as an important medical food, and some of the active constituents possess anti-atherosclerotic properties [89,90], anti-angiogenic activity [83], and anti-tumor activity [91]. Its beneficial effects in the CNS were also documented [80,82,92,93,94,95,96]. 

**Figure 2:**
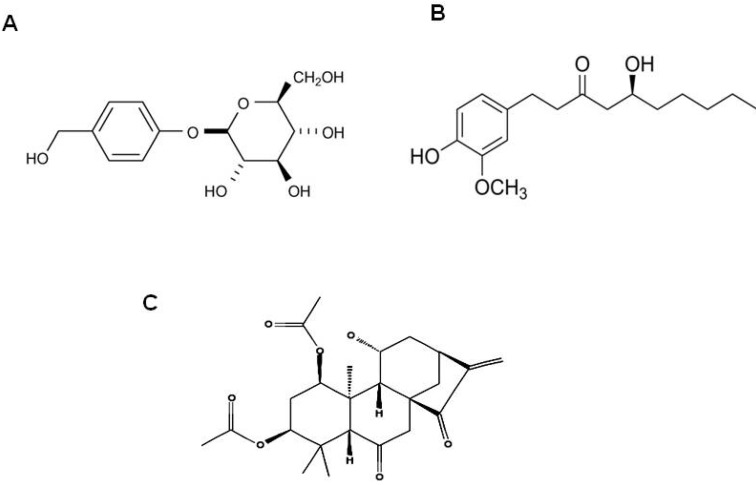
The molecular structure of gastrodin (A), 6-gingerol (B) and inflexin (C).

Regarding its neuroprotective effects, gastrodin (Figure 2A) exerts beneficial actions making it useful for treatment of dizziness, epilepsy, stroke and dementia [78,97]. Gastrodin and *para*-hydroxybenzyl alcohol facilitate memory consolidation and retrieval as shown in the passive avoidance task test in rats [92]. The aqueous extract of GE improves D-galaxies-induced memory impairment in mice and performance on a step-down, passive-avoidance task in senescent mice. Based on these reports, GE could be further developed as a potential therapeutic candidate for treating microglia activation-mediated neuroinflammatory and neurodegenerative diseases.

## 8. Gingerol from *Zingiber officinale*

Ginger, the rhizome of the plant *Zingiber officinale,* has a long history of medicinal use. In traditional oriental medicine, ginger has been used to treat a wide range of ailments including stomach aches, diarrhea, nausea, asthma, respiratory disorders, toothache, gingivitis, and arthritis [98,99,100]. Several studies have shown that ginger inhibits pro-inflammatory cytokines, including IL-1β, IL-2, IL-12, TNF-α, and interferon (IFN)-gamma [101]. Ginger also has been shown to decrease synthesis of pro-inflammatory prostaglandins and leukotrienes via inhibition of COX-2 and 5-lipoxygenase (5-LOX) enzymes, which are the targets for numerous anti-inflammatory pharmaceuticals.

Grzanna *et al.* tested the effects of a ginger extract on THP-1 monocytic cells to determine whether it can block the induction of pro-inflammatory cytokines in these cells stimulated with LPS. The results of this study suggest that the anti-inflammatory properties of the ginger extract may provide beneficial effects similar to those of currently used COX inhibitors [102]. 

Recently, Jung *et al.* reported that the hexane fraction of *Zingiberis Rhizoma* Crudus extract inhibits the production of nitric oxide and pro-inflammatory cytokines in LPS-stimulated BV-2 microglial cells via the NF-κB pathway [103]. The authors indicated that ginger hexane extract significantly inhibited the excessive production of NO, PGE_2_, TNF-α, and IL-1β in LPS-stimulated BV-2 cells. Ginger extract also attenuated the mRNA expressions and protein levels of iNOS, COX-2, and pro-inflammatory cytokines. The molecular mechanisms that underlie ginger hexane extract-mediated attenuation of neuroinflammation were related to the inhibition of the phosphorylation of three mitogen-activated protein kinases (MAPKs), extracellular signal-regulated kinases 1 and 2 (ERK1/2), p38 MAPK, and c-Jun N-terminal kinase (JNK), and the activation of NF-κB [103]. 

6-Gingerol (Figure 2B), one of the active ingredients of ginger, has been reported to impart ginger with its anti-inflammatory properties. The 6-gingerol inhibited the production of pro-inflammatory cytokines from LPS-stimulated macrophages, and inhibited COX-2 expression by blocking the activation of p38 MAP kinase and NF-κB in phorbol ester-stimulated mouse skin [104,105]. Data indicate that several doses of 6-gingerol selectively inhibit production of pro-inflammatory cytokines such as TNF-α, IL-1, and IL-12 by murine peritoneal macrophages in the presence of LPS stimulation. The authors also revealed that 6-gingerol does not affect antigen presenting cell (APC) function or cell surface expression of MHC II and co-stimulatory molecules [105]. These remarkable beneficial properties of ginger and 6-gingerol and the lack of gastrointestinal and renal side effects distinguish it from other NSAIDS. Considering the broad spectrum of ginger’s anti-inflammatory actions and its safety record in clinical trials, it is likely to be a valuable dietary supplement in the treatment of neurodegenerative and neuroinflammatory diseases. However, the ability of gingerol to cross blood-brain barrier has not yet been explicitly demonstrated and needs further investigation. 

## 9. Obovatol from *Magnolia Obovata*

Obovatol is the main component of leaves of *Magnolia obovata*, a medicinal plant that has long been used as a folk remedy in East Asia. Previous studies showed various biological activities of obovatol such as anti-bacterial, anti-tumor, and anti-platelet effects [106,107,108]. The anti-inflammatory activity of obovatol was also reported in LPS-stimulated mouse macrophage RAW264.7 cells [109]. With respect to CNS-related activity, obovatol reportedly has anxiolytic effects that are mediated by GABA-benzodiazepine receptors-activated Cl^-^ channel opening [110]. Most recently, the effects of obovatol on microglial activation and neuroinflammation were examined using cultured cells and a mouse model of neuroinflammation [111]. The study showed that obovatol inhibited inflammatory activation of microglia *in vitro* and neuroinflammation *in vivo*, and the compound exerted protective effects against microglia-mediated neurotoxicity. However, no direct evidence regarding its ability to cross blood-brain barrier has been reported. Based on the affinity chromatography followed by liquid chromatography and tandem mass spectrometric analysis, the authors identified peroxiredoxin 2 (Prx2) as the molecular target of obovatol, implicating microglial redox regulation in the inflammatory neurodegeneration. The report also suggested that Prx2 is a novel drug target that can be exploited for the therapeutic modulation of microglia activation and neuroinflammation.

## 10. Other Natural Products and Components

Inflexin is a compound derived from the natural plant *Isodon excisus* (Max.) Kudo (Labiateae), which is a perennial herb distributed widely in Korea, China, and Japan (Figure 2C). The aerial parts of this plant have been used for detoxification and treatment of gastrointestinal disorders [112], anorexia, indigestion, stomach ache, inflammation, and esophageal carcinoma [113]. Studies from our lab and others revealed that inflexin has the potential to inhibit LPS-induced NF-κB activation in RAW264.7 macrophage cells [114] and in LPS-treated BV-2 microglial cells [115]. In the later study from our laboratory, inflexin significantly inhibited the release of nitric oxide (NO). Both the mRNA and the protein levels of iNOS were decreased in a concentration-dependent manner. Inflexin also inhibited the expression of COX-2, and effectively reduced the LPS-induced expression of pro-inflammatory cytokines in a dose-dependent manner. Furthermore, inflexin inhibited the degradation of IκB-α and the activation of NF-κB p65 subunit and Akt [115]. Due to its potent beneficial action in the inhibition of microglial activation, this compound could give rise to stable derivatives for the treatment of microglia-mediated neuroinflammation and could be developed as a potent therapeutic agent in treating various neuroinflammatory diseases.

**Figure 3:**
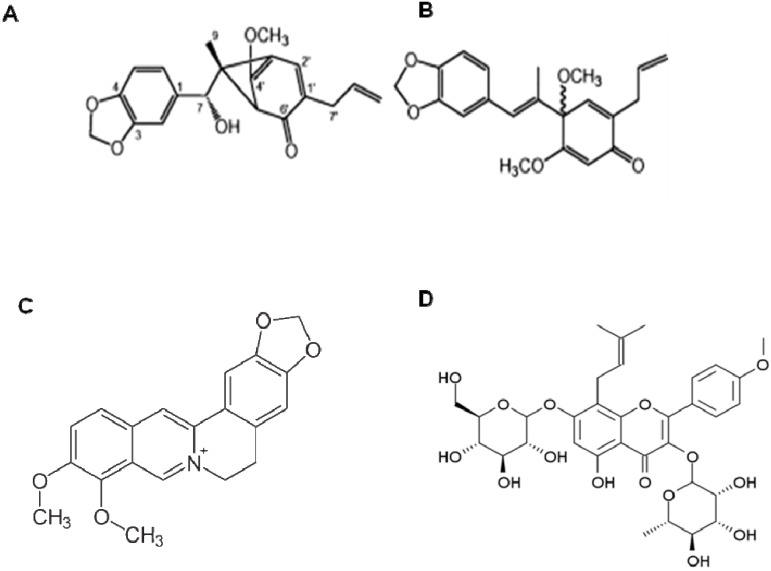
The molecular structure of neolignans (A and B), berberine (C) and icariin (D).

*Piper kadsura* Ohwi (Piperaceae) is a medicinal vine-like plant distributed in the coastal forest regions of Korea, Japan, Taiwan, and China where it covers rocks and trees. The stem part of this plant, known as haifengteng, is widely used in the herbal medicinal prescriptions for the treatment of asthma and arthritic conditions. Recently, the neolignans (Figure 3A and Figure 3B) isolated from *Piper kadsura* were shown to possess strong anti-neuroinflammatory activity as demonstrated by a reduction in nitric oxide (NO) production in LPS-activated BV-2 microglial cells [116].

Another natural product *Ganoderma lucidum* (GL) protects against dopaminergic neuron degeneration through inhibition of microglial activation. GL extracts significantly prevent the production of microglia-derived pro-inflammatory and cytotoxic factors (nitric oxide, TNF-α, and IL-1β), and down-regulate the TNF-α and IL-1β gene expressions at the mRNA level as well, indicating that GL may be a promising agent for the treatment of neuroinflammatory diseases [117].

Blueberries are flowering plants of the genus *Vaccinium*. Blueberries have a diverse range of micronutrients, with notably high levels of the essential dietary mineral manganese, vitamin B6, vitamin C, vitamin K and dietary fiber. Blueberries contain anthocyanins, other antioxidant pigments, and various phytochemicals that may reduce the risks of inflammatory disease and certain cancers [118,119]. Recently, blueberry has been reported to oppose *β*-amyloid peptide-induced microglial activation via inhibition of p44/42 mitogen-activation protein kinase in murine primary microglial culture. Also, blueberry significantly enhances microglial clearance of A*β*1–42, inhibits aggregation of A*β*1–42, and suppresses microglial activation, all via suppression of the p44/42 MAPK pathway [120].

Berberine (Figure 3C), one of the major constituents of the Chinese herb *Rhizoma coptidis*, suppresses neuroinflammatory responses through AMP-activated protein kinase activation in BV-2 microglial cells [121]. 

*Epimedium brevicornum* Maxim (Berberidaceae) is an important traditional herbal medicine originally used in ancient China for its tonic effect. Icariin (ICA, C_33_H_40_O_15_, MW: 676.65; Figure 3D), with the chemical name 2-(4′-methoxylphenyl)-3-rhamnosido-5-hydroxyl-7-glucosido-8-(3′-methyl-2-butylenyl)-4-chromanone, is a major component isolated from *E. brevicornum*. As a highly interesting natural flavonoid compound for drug development, icariin has a broad spectrum of established pharmacological functions, including antioxidant [122], immunoregulatory [123], and antidepressant-like effects [23], stimulation of angiogenesis [124], and induction of cardiomyocyte differentiation [125]. Recent studies have reported that icariin inhibits the inflammatory response by decreasing the production of TNF-α, IL-6, NO and adhesion molecules (CD11b) both in the RAW264.7 macrophage cell line and in mouse serum [126]. In addition, icariin also exhibits an anti-inflammatory effect on LPS-treated murine chondrocytes through inhibition of NO and MMP synthesis [127]. A recent report by Zheng *et al*. revealed that Icariin attenuates LPS-induced microglial activation by inhibiting TAK1/IKK/NF-κB and JNK/p38 MAPK pathways. These findings provide mechanistic insights into the suppressive effect of icariin on LPS-induced neuroinflammatory response in microglia, and emphasize the neuroprotective effect and therapeutic potential of icariin in neuroinflammatory diseases [128].

**Figure 4:**
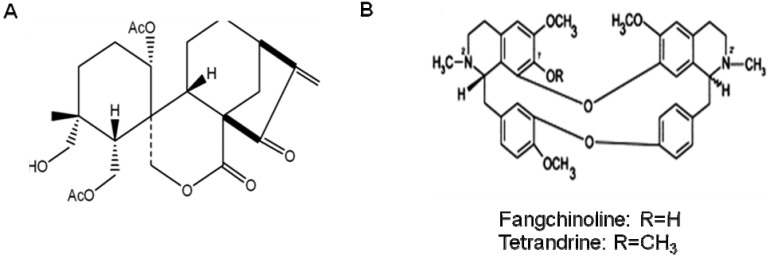
The molecular structure of isodojaponin D (A), fangchinoline and tetrandrine (B).

*Isodon japonicus* (Burm.) Hara (Lamiaceae) is a perennial plant with wide distribution in Korea, China, and Japan. The aerial parts of *I. japonicus* have commonly been used as traditional folk medicines for treatment of gastrointestinal disorders, tumors, and inflammatory disease [129]. Sequential column chromatography of *I. japonicus* has allowed isolation of a new ent-kaurane type diterpene, isodojaponin D (19-hydroxy-1α, 6-diacetoxy- 6, 7-seco-ent-kaur-16-en-15-one-7, 20-olide; Figure 4A) [130]. Earlier reports indicated that isodojaponin D has neuroprotective effects against β-amyloid-induced toxicity [116]. Recently, isodojaponin D was reported to inhibit LPS-induced microglial activation [131]. Isodojaponin D significantly decreased the LPS-induced production of COX-2 and iNOS. In addition, LPS-induced pro-inflammatory cytokines, including IL-1β, IL-6, and TNF-α, were also decreased through NF-κB and MAPK signaling pathways. The reports suggest that isodojaponin D could play a beneficial role in the treatment of neurodegenerative and neuro-inflammatory diseases.

Furthermore, the anti-inflammatory constituents tetrandrine and fangchinoline (Figure 4B) found in *Stephania tetrandra* have been shown to decrease IL-1β, IL-6, IL-8 and TNF-α [132], and to decrease leukotriene and prostaglandin generation [133]. More importantly, tetrandrine has been shown to inhibit the production of TNF-α and IL-6 by microglial cells [134], which damage nerve cells. Similarly, *Urtica dioica*, also known as “stinging nettle”, reduces IL-1β, IL-2, IFN- , and TNF-α [135,136]. Supplementation of stinging nettle in humans has been shown to decrease LPS induction of inflammatory mediators, triggering an 80% reduction in TNF-α and a 99% reduction in IL-1β [137].

Several flavonoids derived from plant origin such as wogonin, tectorigenin, tectoridin, apigenin, luteolin, and fisetin modulate microglial activation at the cellular level and exert their anti-neuroinflammatory properties, suggesting their importance as a potent therapeutic agent for treating neuroinflammatory diseases [14,138,139,140,141,142]. In particular, fisetin (3,3,4,7-tetrahydroxy flavone), a naturally occurring flavonoid commonly found in strawberries and other fruits and vegetables, inhibits neurotoxic microglial activation. Studies have also demonstrated that fisetin exhibits a wide variety of biological activities, including anti-cancer [143], neurotrophic [144], anti-oxidant [145], and anti-inflammatory effects in mast cells [146]. Along with the anti-inflammatory effects of fisetin in peripheral mast cells, its anti-inflammatory effect in brain microglia has recently been investigated [147]. Fisetin inhibits the production of inflammatory mediators, and suppresses NF-κB and p38 mitogen-activated protein kinase activation in LPS-stimulated mouse microglia cells. In addition, the flavonoid exhibits a neuroprotective effect by attenuating microglial neurotoxicity in the microglia-neuron co-culture model.

## 11. Conclusions

A growing body of evidence indicates that neuroinflammation may contribute to the neurodegenerative process. It is believed that activated microglia cells, which compose the majority of this inflammatory response, contribute to the neurodegenerative process. Suppression of microglia activation may provide an effective therapeutic intervention that alleviates the progression of the neurodegenerative diseases. Recently, the natural products and their components have received considerable attention as alternative candidates for therapeutic purposes. They have a reputation for being safe, inexpensive, and readily available. However, only a few substances have been studied in depth, and much of the work is in the preliminary stages. It remains be adequately studied clinically whether the therapeutic effects indicated by the experimental settings are similar in humans. If these substances have benefits for humans that are reflected in the laboratory studies, then moderate doses consumed regularly over years might be able to prevent or at least slow the development of the disease. Continued investigation of the mechanisms underlying microglial activation, regulation of neuroinflammation, and the modulatory role of natural products and their components could not only lead to the discovery of novel neuroprotective agents, but also help us to understand the complex pathophysiology of neurodegenerative diseases.
